# The #MeToo Movement in the United States: Text Analysis of Early Twitter Conversations

**DOI:** 10.2196/13837

**Published:** 2019-09-03

**Authors:** Sepideh Modrek, Bozhidar Chakalov

**Affiliations:** 1 Health Equity Institute San Francisco State University San Francisco, CA United States; 2 Economics Department San Francisco State University San Francisco, CA United States

**Keywords:** social media, sexual abuse, sexual assault, machine learning, infodemiology, infoveillance

## Abstract

**Background:**

The #MeToo movement sparked an international debate on the sexual harassment, abuse, and assault and has taken many directions since its inception in October of 2017. Much of the early conversation took place on public social media sites such as Twitter, where the hashtag movement began.

**Objective:**

The aim of this study is to document, characterize, and quantify early public discourse and conversation of the #MeToo movement from Twitter data in the United States. We focus on posts with public first-person revelations of sexual assault/abuse and early life experiences of such events.

**Methods:**

We purchased full tweets and associated metadata from the Twitter Premium application programming interface between October 14 and 21, 2017 (ie, the first week of the movement). We examined the content of novel English language tweets with the phrase “MeToo” from within the United States (N=11,935). We used machine learning methods, least absolute shrinkage and selection operator regression, and support vector machine models to summarize and classify the content of individual tweets with revelations of sexual assault and abuse and early life experiences of sexual assault and abuse.

**Results:**

We found that the most predictive words created a vivid archetype of the revelations of sexual assault and abuse. We then estimated that in the first week of the movement, 11% of novel English language tweets with the words “MeToo” revealed details about the poster’s experience of sexual assault or abuse and 5.8% revealed early life experiences of such events. We examined the demographic composition of posters of sexual assault and abuse and found that white women aged 25-50 years were overrepresented in terms of their representation on Twitter. Furthermore, we found that the mass sharing of personal experiences of sexual assault and abuse had a large reach, where 6 to 34 million Twitter users may have seen such first-person revelations from someone they followed in the first week of the movement.

**Conclusions:**

These data illustrate that revelations shared went beyond acknowledgement of having experienced sexual harassment and often included vivid and traumatic descriptions of early life experiences of assault and abuse. These findings and methods underscore the value of content analysis, supported by novel machine learning methods, to improve our understanding of how widespread the revelations were, which likely amplified the spread and saliency of the #MeToo movement.

## Introduction

Public discourse on sensitive topics, ranging from sexual violence to health (mis)behaviors, increasingly occurs on social networking platforms such as Twitter [1-3]. Public health officials and social scientists alike are turning to Twitter to better understand who participates in these conversations, what new information may be gleaned, and the reach of online messages. In health fields, studies of the content of social media posts have a variety of aims, including producing new data to support surveillance efforts, predicting onset of a variety of conditions, and targeting interventions [4]. Detailed content analyses of posts, above and beyond search frequencies and hashtag analysis, have examined health issues such as influenza, allergies, and a variety of mental health conditions (depression, postpartum depression, eating disorders, etc) [5-10]. Likewise, social scientists have conducted detailed content and network analysis of social media posts to understand salient topics in particular networks (Black Twitter and online Feminism) and the reach of salient topics [11-14].

The recent explosion in the public discourse on sexual violence is an interesting case to consider. Sexual violence, including sexual harassment, abuse, and assault, is highly pervasive and has long-term behavioral and mental health sequelae [15]. In the US, one in three women experience unwanted sexual contact in their lifetime [16]. Although hashtag movements such as #BeenRapedNeverReported had traction and encouraged public disclosures of personal experiences of rape [12], these initial movements were relatively small. The public discourse on sexual violence changed substantially on October 15, 2017, when actress Alyssa Milano called her followers to post “MeToo” if they had ever experienced unwanted or inappropriate sexual contact [5].

Ms Milano’s tweet immediately went viral, with 1,595,453 tweets posted in the first week, and ignited a movement where victims of sexual assault, abuse, and harassment felt empowered to divulge as much, or as little, information as they wanted about their personal experiences. The phrase “MeToo” was coined by Tarana Burke, a civil rights activist, as a way to raise awareness and provide support for survivors of sexual violence. Ms Milano’s use of this phrase in her tweet, as opposed to other more explicit hashtags such as #BeenRapedNeverReported, allowed posters to retain some privacy about the details of the event and still participate. This led to millions of users joining the conversation and subsequent normalizing of the revelations. Meanwhile, the wide use of the generic #MeToo encouraged an outpour of detailed revelations. Because of the massive size of the #MeToo movement, many who may not have experienced sexual violence were confronted with the knowledge that members of their network had.

In this study, we aim to describe the public disclosures of sexual violence within the first week of the #MeToo movement. Here, we document the content, quantify the scale, and present the demographic characteristics of Twitter users who disclose incidents of sexual assault/abuse in the early conversation on Twitter for the #MeToo movement. We use simple machine learning tools to create the archetype of tweets, which often included detailed accounts of sexual assault and abuse and early life experience of such events among women from all walks of life. Next, we categorize the content of individual-level tweets to estimate the proportion of all #MeToo tweets with such revelations in the first week of the movement. Furthermore, we use our categorization to detail the demographic characteristics of posters with revealed events and the reach of the revealed events on the Twitter platform generally.

## Methods

### Data

The data for this project are tweets, short messages of 140 characters or less, sent from a Twitter user (Twitter handle) to their network of followers. Twitter data are considered existing data in the public domain and therefore exempt from human subject review.

**Figure 1:**
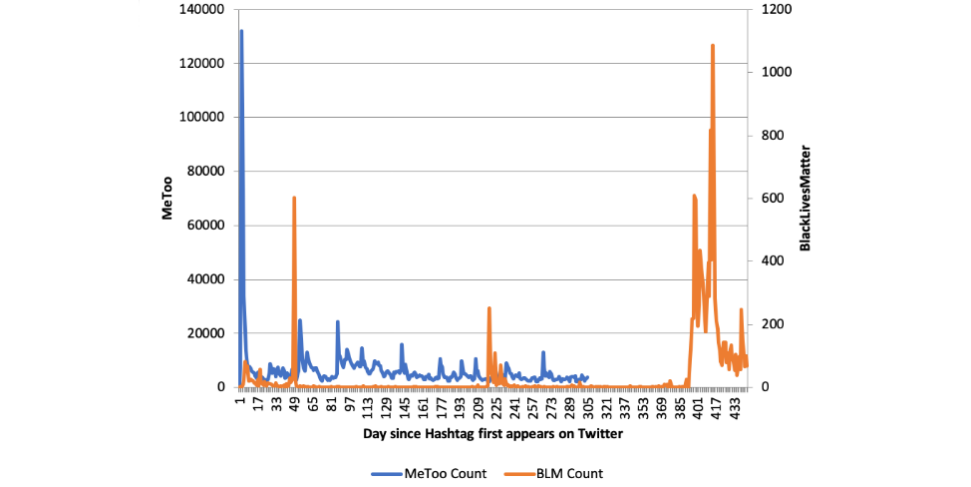
Comparison of relative time patterns of novel English tweets including MeToo and BlackLivesMatter. MeToo counts are on the left axis, and BlackLivesMatter counts are on the right axis. BLM: BlackLivesMatter.

We applied for and were granted access to the Twitter Premium application programming interface (API) platform, which allows users to purchase and query all nondeleted public tweets since the first tweet posted in 2006. We purchased both the counts and full tweets from historical Twitter data between October 14 and 21, 2017 (ie, the first week of the movement). Unlike other social media movements that took several months to take hold, the #MeToo movement had the greatest activity in the first week (Figure 1), which is one reason we limited our data collection to this period. For our analysis, we concentrated only on novel, or user generated, English language tweets with “MeToo” in the text. Novel tweets exclude replies to other’s tweets, retweets without comments, and links to other sites or images. This was chosen to capture posts that were tied to the specific user and that would be available to all of one’s followers. We further limit the tweets to those with geotagged information, placing the tweet in the United States (N=12,337; this count is subject to some variability, as it depends on the day of the query and only includes nondeleted tweets).

Figure 1 shows the daily counts of novel English Language tweets starting from one day prior to the creation of each hashtag on Twitter (July 13, 2013, for BlackLivesMatter and October 14, 2017, for MeToo). The counts derived from the Twitter Premium API vary by the date of query. These counts were extracted on August 14, 2018.

**Figure 2:**
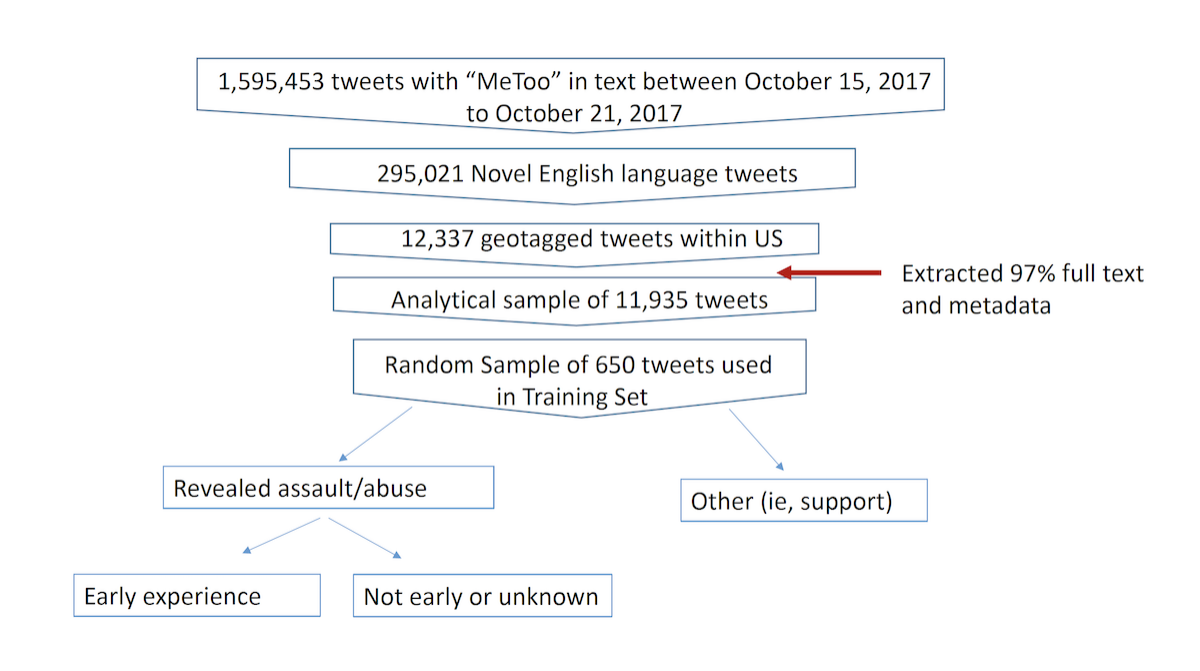
Data flow chart. LASSO: least absolute shrinkage and selection operator; SVM: support vector machine.

The analytic sample for our content analyses comprised the full text and associated metadata for 97% of novel English language US-based tweets in the study period (N=11,935). Figure 2 presents a flowchart of the filtering process on the sample of tweets captured and analyzed (see Multimedia Appendix 1 for details of the selection process).

### Analyses

We first present evidence that the sample of novel English #MeToo tweets in the United States, which we selected for analysis, was comparable in terms of time trends to all novel English #MeToo tweets. We graphed the number of #MeToo tweets per hour starting on October 14, to show the time trends comparing all #MeToo tweets, novel English #MeToo tweets, and US-based geotagged novel English language #MeToo tweets. We also report quantitative measures of correlation on the number of tweets in each of these categories by hour (the Pearson correlation and coefficient of determination).

### Annotation Process

Before analyzing the data, both authors reviewed approximately 2000 tweets from the first week of the movement. Based on this initial review, we chose to focus the analysis on first-person revelations of sexual assault and abuse and childhood experience of sexual assault and abuse. The tweets reviewed revealed several categories of comments with #MeToo hashtags. They included (1) tweets with #MeToo that were support statements (ie, “How many women not in the spotlight have #MeToo #Notaceleb #StillAStar. Proud of them! As Bosch says- ‘everybody counts or nobody counts’”), (2) statements with ambiguous revelations of events (ie, “MeToo” alone and “Kept it buried down for many years and didn't even really realize how it impacted me.”), (3) statements with detailed revelations of events (ie, “I was sexually assaulted by a family member when I was 8 the trauma *[sic]* it causes never ends, remember that before you ruin someone's life”), and (4) others (ie, either negative comments, unrelated content, or riding off the hashtag; “Not watched any games this weekend @NFL @nflcommish @nflnetwork #metoo didnt buy nfl merchandise this year Got refund for #NFLSundayTicket”).

**Figure 3:**
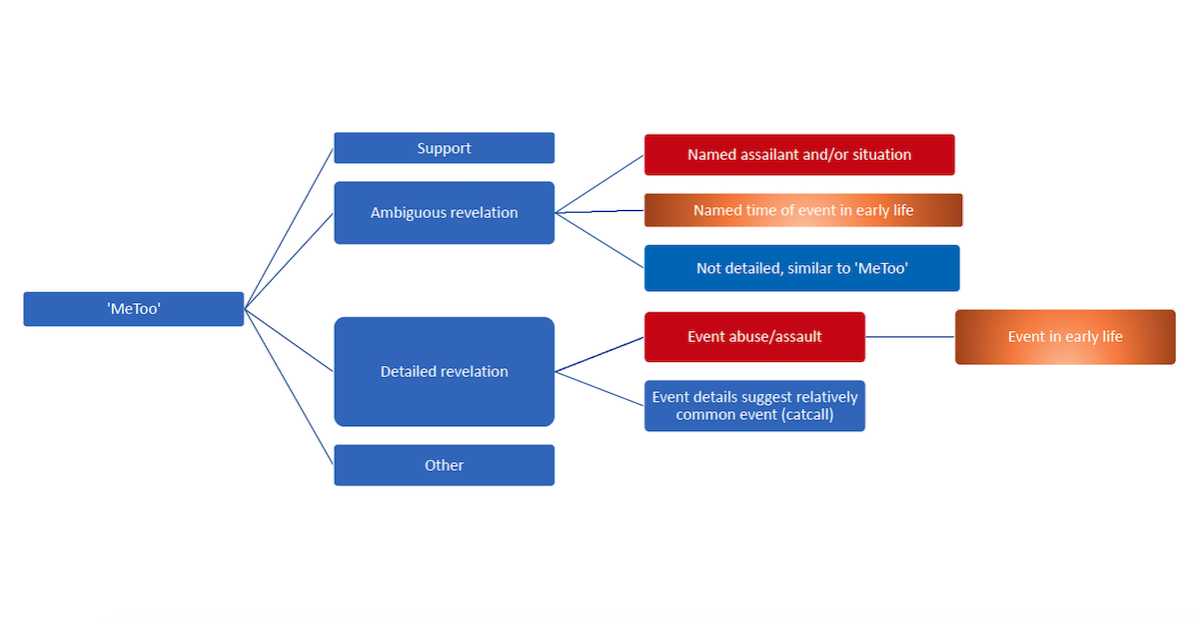
Classification flow chart.

Based on this initial review, we chose to focus on the second and third categories and created an annotation rubric to categorize first-person revelations. For ambiguous statements (ie, category 2) where it was clear an event occurred, such as a revelation with an alleged assailant’s name or a situation but little details on the actual actions, we decided that this recollection likely reflects enduring trauma and should be categorized as a revealed abuse or assault. If the ambiguous events were experienced in childhood, it would be classified as an early life experience of abuse or assault (“I was a child too scared to speak. Till this day, it still haunts me”). If the statement was too ambiguous, did not make references to childhood, or provided little detail, we did not classify it as a case of abuse (ie, “Sometimes the stories we don't share are the ones that affect us and continue to scare us the most” and “Tried to tweet about my #metoo moment and deleted it bc it still feels like my fault. The voices you aren’t hearing in this are deafening”). For the third category—the clearest case—based on the purported event, we categorized the revelations as abuse or assault and early life experience based on the details provided. Figure 3 provides a schematic of our annotation process.

We randomly selected a subset of 650 tweets from the novel English US-based #MeToo tweets as the training set for the analysis. Based on our rubric, both authors classified the 650 randomly selected tweets. Each tweet was categorized along two main dimensions: if the tweet revealed details of an experience of sexual assault and abuse and if the details suggest that the event happened in early life. All other types of tweets (support, too ambiguous, or other) were categorized as not revealing an incident of abuse or assault. We used an approximate age cut off of 22 years (or references to college or earlier schooling) to distinguish and delineate early life experience. This was chosen to capture college experience under the category of early life. All other tweets were categorized as “other.” The concordance between the two authors’ categorizations was 94% for sexual abuse and assault and 98% for childhood experience. Most disagreements on the abuse/assault categorization were on cases with ambiguous revelations, which could be argued either way. Most disagreements on childhood experience were in cases where the revealed event was placed in the past. Many cases point to an event in the distant past, but it was hard to ascertain the age of the poster at the time of the revealed event, because we did not have the current age of the poster. Given these minor differences in interpretation, we decided to use one author’s (BC) categorization in the training set. To assess our model performance, we used the other author’s (SM) classification in the test set for calculating positive and negative predictive values.

Tweets were categorized along two dimensions: if they disclose an experience of sexual assault and abuse (red in Figure 3) and if the details suggest that the event happened in early life (orange in Figure 3). All other tweets were categorized as “other.” Multimedia Appendix 2 provides examples of the categorizations performed by the authors, which were guided by the categorizations in the “Rape Culture Pyramid” graphic version 1 created by Ranger Cervix and Jaime Chandra in mid-2016 [17].

### Prediction Methods

To provide exemplars of the disclosed tweets, we used simple supervised machine learning methods— least absolute shrinkage and selection operator (LASSO) regression models—on the training sample to find most predictive words for classification along both dimensions [18]. This allows us to present the most predictive words, which serve as an archetype of the tweets with such revelations.

LASSO regressions are a common tool for economists who are using text as data and performing simple computational linguistic analyses [19]. The LASSO, a penalized linear model with L1 penalization, serves as a shrinkage method to help perform feature/word selection to identify the most predictive words from a list of candidate words in a supervised learning environment. These methods are recommended because they are intuitive and interpretable. LASSO regression was chosen over ridge regression, another penalized linear models with L2 penalization, because it is more efficient for variable selection. Furthermore, LASSO was chosen over elastic net regression, which is sometimes preferred when there is substantial correlation between features/words, because we found limited correlation in the words in the training set data.

After removing stop words, we had 11,931 unique words in our training set from 650 tweets. We stemmed each word, examined the list for misspelling, and considered words (and stubs) that appear in at least five tweets to limit the sparsity of our sample, leading to 1186 unique words. From these words, the authors selected 109 words related to sexual abuse and assault. This filtering limited the sparsity of the data further. We then used LASSO regressions on a matrix of 650 tweets coded for sexual assault/abuse (or coded for early life experience) and 109 words, each of which was treated as a binary flag if it appeared in each tweet. For example, if the tweet included the word “Rape” or “Raped” in it, then the variable “Rape” was coded as 1.

The LASSO model minimizes an objective function 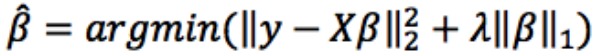, which is a constrained ordinary least squares model (OLS), to find the words with the strongest predictive power on tweets coded as having a revelation of sexual assault or abuse.

Equation 1: 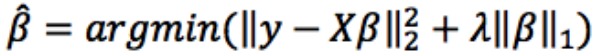, where 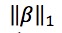
is the L1 Norm of the estimated β coefficients; this is the sum of the absolute value of all the β coefficients, and 
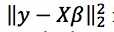
is the sum of squared deviation of the predicted outcome relative to the actual outcome for each observation. This algorithm is the same as an OLS with an added penalization for large estimated β coefficients. The form of the penalization with an L1 Norm drives the magnitude of many β coefficients to zero. This essentially makes the algorithm identify and select features/words that are most predictive [18].

The LASSO models were implemented in the statistical software R ([computer program] Version 3.5.0. Vienna, Austria: R Foundation for Statistical Computing; 2018) using the “glmnet” package. The results of the LASSO model are validated with ten-fold cross-validation. The cross-validation allows us to pick the model with the λ value having the lowest mean squared error or model variance. From this selected model, we obtained a list of the 35 most predictive words for tweets with revelations of sexual assault/abuse words and 34 most predictive words for tweets with revelations of early life experience of sexual assault/abuse words. These word lists were then categorized by the authors.

We then used the same training sample of tweets to train the support vector machine (SVM) models to classify the remaining sample of tweets (N=11,285) along the same two dimensions—experience of sexual assault/abuse and early life experience of sexual assault/abuse [18,20].

An SVM is a discriminative classifier that is used on training data to define a separating hyperplane in multidimensional space and then uses this hyperplane to categorize new data. In the training set, data points that are closer to a potential defining hyperplane (ie, support vectors) are given more weight. The goal of the underlying algorithm is to maximize the margin between the support vector data points and the separating hyperplane using a cost function [18].

In our case, we have a matrix of 11,931 unique words (dimensions) and 650 labeled outcome data. From this information, the algorithm defines a separating hyperplane. Based on this hyperplane, the remaining 11,285 tweets are categorized. To perform the SVM model operations, the package “RTextTools” was used in R software.

We assessed the quality of the SVM model’s classification with four test sets, two for each classification category, to estimate positive and negative predictive values. To calculate the positive predictive value (PPV) and negative predictive value (NPV), we sampled 50 tweets four times as test sets. The first two samples were used to assess PPV and NPV for the model predictions of sexual assault and abuse. The second two samples were used to assess PPV and NPV for the model predictions of early life experience of sexual assault and abuse. Given that BC’s classification was used in the training set, we used SM’s classification on the test set to assess the model.

For each sample of the first two samples, one author (SM) assessed the content of the tweet for a revelation of an experience of sexual assault and abuse. We treated the human-assessed content as the gold standard and calculated the proportion of the time that the SVM algorithm’s classification was the same as the human classification. PPV was calculated as the number of true positives (agreement between human and algorithm) divided by the number of positive cases found by the SVM algorithm. NPV was calculated as the number of true negatives divided by the number of negative cases found by the SVM algorithm. We repeat the process and manually categorize the content of the last two samples for revelations of early life experience of sexual assault or abuse to calculate PPV and NPV

Based on these classifications, we used a previously vetted commercial service, Demographics Pro [21,22], to infer the demographic characteristics of the individuals who revealed an incident of abuse/assault and childhood experience (Multimedia Appendix 1 for description of their algorithm) . We then empirically derived estimates of the 25%-75% range of the number of followers from each poster who revealed an event. This allows us to conduct a crude calculation to capture a lower bound on the reach of the tweets with revelations of sexual assault/abuse in the first week of the movement. This estimate represents the number of potential Twitter users who would have been exposed to revelations of sexual assault/abuse posted by someone they follow.

## Results

**Figure 4:**
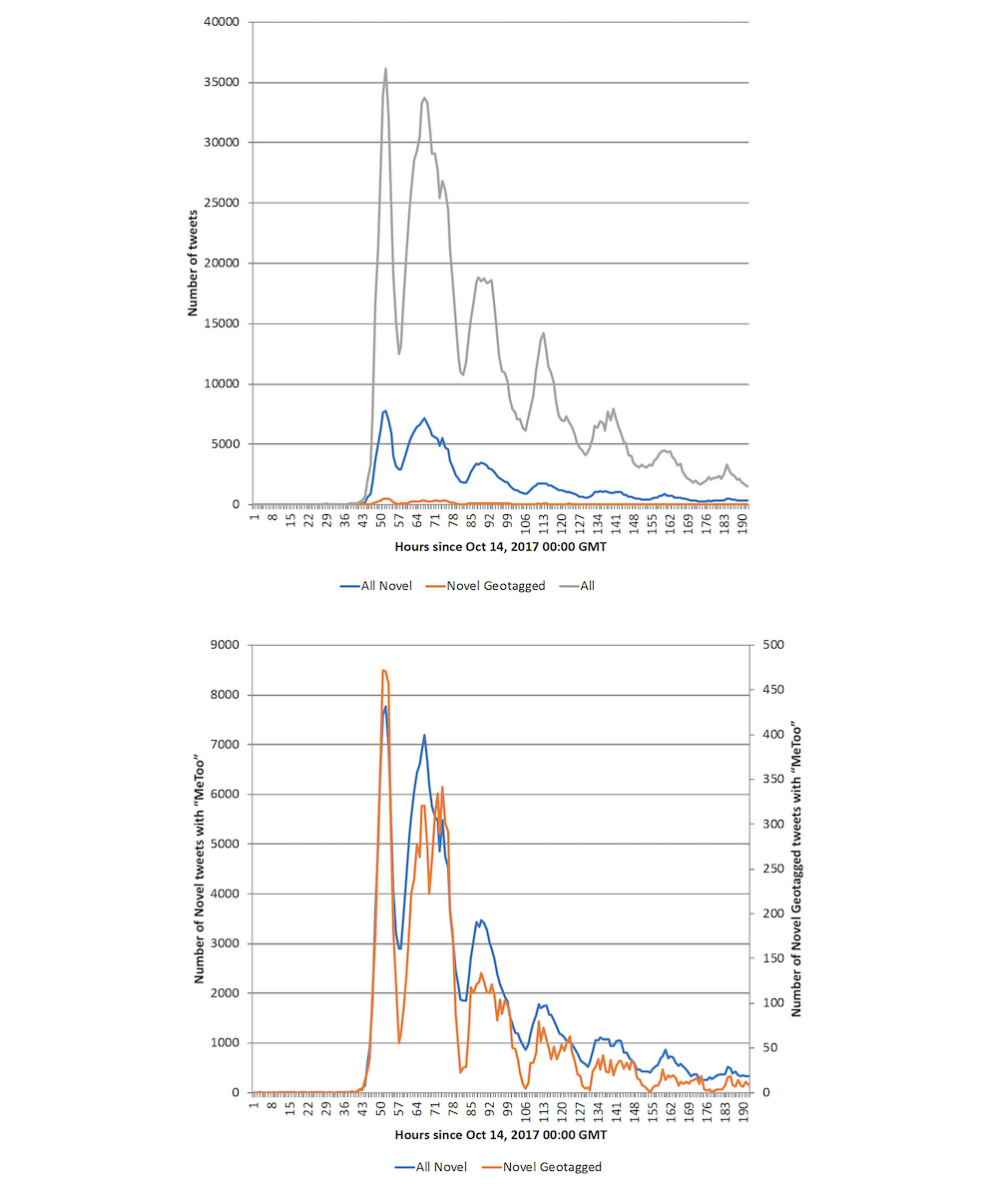
Hourly counts of "MeToo" tweets. Top: Hourly counts of "MeToo" tweets by category (overall, novel English, and geotagged novel English). Bottom: Hourly counts of all novel English language tweets with the phrase "MeToo" and hourly counts of all novel English language geotagged tweets in the United States.

Figure 4 shows the time trends of #MeToo tweets, illustrating how the movement grew. We presented hourly counts of tweets from October 14, 2017, 00:00 GMT, the day before the movement began. We also compared all #MeToo tweets, the novel English #MeToo tweets, and the novel geotagged English #MeToo tweets—the main sample used in this study—to show that they follow a similar overall time trend in the first week. The Pearson correlation between the counts of hourly #MeToo tweets and novel geotagged English #MeToo tweets was 0.96, and the coefficient of determination was 0.92. The counts derived from the Premium API in Figure 4 vary by the date of query. These counts were extracted on June 12, 2018.

Author BC’s classification of the 650 randomly selected tweets in the training set revealed that approximately 19% of these tweets included a first-person revelation of sexual assault/abuse. BC’s classification was used in supervised machine learning methods with LASSO regressions to identify the most predictive words. Textbox 1 summarizes and organizes the most predictive words related to revelations of sexual abuse/assault. The most predictive words are organized into descriptions of time, persons, actions, body parts, and words indicating state and other. For example, the predictive actions verbs include “Rape,” “Grope,” “Grab,” and “Shout” among others. Other highly predictive words relate to an intoxicated state (“Drunk” or “Drug”). Many predictive words placed the revealed event in the past, particularly in early life (“Age,” “College,” “First Time,” and “Years Old”).

**Words contained in Tweets that consistently predict a revelation of sexual abuse or assault (most predictive words categorized).**

**Time/age**

- Age
- College
- First time
- Fifteen
- Grade
- Hasnumber (Hasnumber is an overall indicator of whether there is a number in the text.)
- Kindergarten
- Old
- Year
- Years ago
- Years old

**Person**

- Boyfriend
- Coworker
- Date
- Father
- Man
- Rapist
- Police
- Stranger
- Teacher
- Uncle

**Action**

- Advantage
- Chase
- Grab
- Grope
- Rape
- Shout

**Body Part**

- Arm
- Boob
- Butt

**State**

- Drug
- Drunk

**Other**

- Daylight
- Door
- Interview
- Frat (This term can indicate a location, time/age, or an implied level of intoxication. The term frat can indicate a time (college is when most people go to frats), a place such as a frat house, and a level of intoxication because frats are often where alcohol is served.)

Of the 650 randomly selected tweets in the training set, the authors categorized 5% of the tweets as having indicated an early experience of sexual assault/abuse. Textbox 2 summarizes and organizes the most predictive words related to early experience of sexual abuse or assault. Although many of the predictive terms are the same, the list of persons, or possible assailants, listed for those who had an early life experience include terms like “Neighbor” and “Step-Father.” In addition, the states of being “Asleep” and “Afraid” are highly predictive. Together, these predictive words provide a picture of the types of tweets posted during the early phase of the movement. Note that predictive words are different from a word cloud that captures the frequency of a word. For example, “MeToo” was in every one of the tweets we examined but was not, and could not be, predictive of a disclosure of sexual assault/abuse, given our method. The word “Grope,” on the other hand, was not in every tweet and was a predictive word in tweets with revelations of assault/abuse.

Next, we use the SVM algorithm on the same 650 classified tweets in the training set. Of the remaining 11,285 tweets, the SVM algorithm categorized 1287 tweets (11.4%) as having a revelation of sexual assault/abuse and 657 tweets (5.8%) as having a revelation of early sexual assault/abuse (Multimedia Appendix 3). To assess the validity of the SVM classification, we calculate both the PPV and NPV in comparison with human categorized test sets. The SVM classification for sexual assault/abuse had a PPV of 87%, meaning that there is 87% concordance between the algorithm and our manual assessment of the tweets with revelation of sexual assault/abuse. The NPV was 83%, suggesting that the algorithm may have slightly underreported the number of tweets with revelations of sexual assault/abuse in comparison to the authors’ assessment. For early life experience classification, the PPV is 79% and the NPV is 95%. There was a slightly lower concordance between the algorithm and the authors’ assessment of tweets identified as revealing an early life experience of sexual assault/abuse, but the false negative rate was quite low. Together, these assessments of the algorithm suggest that our algorithm performed well and would be highly congruent with the human-generated classifications.

In Table 1, we present the percentage of daily tweets with revelations of sexual assault/abuse and early life experience of sexual assault/abuse based on the SVM classification. In the first 2 days of the movement (October 15-17), 11%-13% of tweets revealed an experience of sexual abuse or assault and over half of the tweets revealed that the experience of sexual assault/abuse occurred in early life. As the movement carried forward, the revelations of sexual assault/abuse decreased to about 6% of MeToo tweets in the last day of the first week, but the relative proportion with early life experience of these events increased to over 80%, suggesting that more traumatic events were being shared.

Based on the SVM classification, we then used the Demographics Pro prediction service to understand and compare the demographics of posters of sexual assault/abuse during the early #MeToo movement. We present these demographics in Table 2. Based on the results of the SVM model, we identified 1168 unique posters/Twitter handles that revealed an incident of sexual assault/abuse and 612 unique posters that revealed an early life experience of sexual assault/abuse. We shared these Twitter handles with Demographics Pro, who then gave us aggregated predictions on the distribution of the demographic characteristics of these posters. We found that 90% of these Twitter users who posted about their experience of sexual assault/abuse were women. This is congruent with national estimates that 90% of sexual assault victims are women [23]. We also found that white women were overrepresented in the early conversation in our Twitter data with regard to their population in the United States, their proportion as Twitter users, and national estimates of those who report experiencing sexual assault (data not shown). The age distribution also shows that older users of Twitter, aged 25-50 years, disproportionately revealed events.

Finally, from the SVM classification, we used the provided metadata on the number of followers of posters who revealed sexual assault/abuse in the first week of the #MeToo movement to better understand the reach of such revelations. Based on posters’ follower count, we took the 25%-75% range of followers from users with such experiences and calculated a lower bound range on the number of Twitter user who may have seen a first-person revelation. This simplistic calculation provided a range of 5,955,342 to 34,251,628 Twitter users for the reach, which we believe is a substantial lower bound (Multimedia Appendix 3).

We believe the reported reach is an underestimate, because we did not include replies or retweets and our algorithm has a higher false negative rate. We did not examine the network of people who reveal events, which could be highly overlapping and would mean that many users would have seen multiple tweets with revelations. Furthermore, the distribution of followers who posted about sexual assault does not seem to vary substantially from the distribution of followers for Twitter users overall (Multimedia Appendix 4). Finally, we only captured posts on Twitter. Many were also posting #MeToo posts on other social media platforms such as Facebook.

**Words contained in Tweets that consistently predict a revelation of an early experience of sexual abuse or assault (most predictive words categorized).**

**Time/age**

- Age
- College
- First time
- Freshman
- Grade
- Hasnumber (Hasnumber is an overall indicator of whether there is a number in the text.)
- High school
- Kindergarten
- Old
- School
- Years ago
- Years old

**Person**

- Cop
- Coworker
- Date
- Doctor
- Father
- Male
- Neighbor
- Rapist
- Step father
- Teacher
- Uncle

**Action**

- Rape
- Rip
- Screw

**Body part**

- Arm
- Butt
- Pussy

**State**

- Afraid
- Asleep
- Drunk

**Other**

- Concert

**Table 1:** Counts and percent of #MeToo tweets with disclosures of sexual abuse/assault and early experience tweets by date.

Date	Total, n^a^	Abuse/assault, n (%)	Early experience, n (%)
10/15/17	371	43 (11.59)	25 (6.74)
10/16/17	5987	817 (13.65)	420 (7.02)
10/17/17	3174	336 (10.59)	142 (4.47)
10/18/17	1155	113 (9.78)	54 (4.68)
10/19/17	676	57 (8.43)	21 (3.11)
10/20/17	356	31 (8.71)	14 (3.93)
10/21/17	215	14 (6.51)	12 (5.58)

^a^Number of geotagged novel English language tweets in United States.

**Table 2:** Demographic characteristics of abuse/assault and early life experience samples among unique Twitter users.

Characteristic		US census, %^a^	Twitter overall, %^b^	Abuse/assault sample (N=1168), %^b,c^	Early experience sample (N=612), %^b,c^
**Sex**					
	Male	49.2	45.8	10.6	9.2
	Female	50.8	54.2	89.4	90.8
**Age (years)**					
	≤19	25.4	25.02	15.2	13.1
	20-24	6.70	45.33	25.5	24.1
	25-29	7.10	16.10	20	20
	30-34	6.70	7.16	17.8	22
	35-39	6.60	2.40	8.1	7.3
	40-49	12.5	3.25	9.7	9
	50-59	13.3	0.49	2.6	3.7
	≥60	21.7	0.25	1	0.8
**Race/ethnicity**					
	White/Caucasian	60.7	78.7	90.7	89.8
	Hispanic	18.1	7.6	6.2	6.1
	African American	13.4	13.1	2.6	3.3
	Asian	5.8	0.6	0.4	0.8
	Native American/Pacific Islander	1.5	—^d^	—	—

^a^Age distribution based on 2017 American Community Survey 1-Year Estimates (July 1, 2017).

^b^Proportions provided by Demographics Pro on October 18, 2018.

^c^Based on our classification in the analytical sample of geotagged novel English language tweets in the United States.

^d^Not available.

## Discussion

### Principal Findings

We conduct the first quantitative text analysis of the content of the early conversation in the #MeToo movement on Twitter, which was the largest hashtag movement on Twitter in 2017 [24] and the largest public discourse on sexual violence. We use machine learning to provide exemplars of disclosures during the first week of the movement. The most predictive words create an archetype of the content of tweets with the revelation of sexual assault/abuse. They include words that we would expect like “grope” and “rape” as well as states like “drunk,” “asleep,” and “afraid.” Based on our models, 11% of novel tweets in the first week of the movement publicly revealed an experience of sexual assault/abuse and 6% revealed an early life experience of sexual assault/abuse. The initial women sharing were predominately white women aged 25-50 years. Tweets from older posters suggest that these experiences were lodged into these women’s memories and were not inconsequential passing events. Notably, African Americans were underrepresented relative to their Twitter presence in this early conversation. Moreover, given the connectivity possible on Twitter, we estimate that between 6 and 34 million Twitter users may have been exposed to at least one such detailed revelation.

### Limitations

There are key limitations to this study that should be noted. First, based on financial considerations, we could not extract all novel English language posts during the first week of the #MeToo movement. Instead, we chose to focus on those geotagged within the United States. This restriction made the purchase of almost all the tweets that fit this category possible. To examine the representativeness of these tweets, we examined time patterns relative to all #MeToo posts and novel English language #MeToo posts (Figure 3). This analysis suggests that the timing of novel English language geotagged tweets was not different from novel English language tweets. However, if users who allow Twitter to geotag tweets systematically revealed sexual assault/abuse events at a different rate than those who do not allow Twitter to geotag their tweets, then our estimates may be inaccurate. However, we noted that users would have likely enabled geotagging on Twitter at some time prior to their MeToo tweet. Thus, allowing geotagging is not necessarily related to the MeToo tweet. Second, we include only English tweets on the topic of #MeToo even though there were similar movements in different languages (such as #YoTambien or #BalanceTonPorc), and we did note examine tweets outside the United States. For example UK-based or Canada-based English language #MeToo tweets were not included in our data. Again, there could be systematic differences that would affect our estimates, and thus, we acknowledge that our estimates reflect the conversation in the United States. Third, while we examined the time dynamics of many related or counter hashtag movements happening the same week, such as #HimThough, #Ibelieveyou, #Ihearyou, #Iwillnot, and #howIwillchange, we did not include these in our analyses because these hashtags were in response to the #MeToo posts. The content of tweets with these other hashtags were less likely to have revelations of sexual assault/abuse, but rather just voice support. Future analyses could examine who voiced support rather than who revealed events. Fourth, we did not conduct network analysis examining common retweets, which would have had much greater reach. Our focus was on novel revelations that were rarely retweeted relative to support statements, which were often retweeted. Fifth, we only examined the first week of the movement. This was a deliberate choice because the movement went in many directions afterward, with calls to stop posting traumatic events because they triggered women [25] or demands that men post instead of women having to post and relive their trauma. Finally, many tweets were ambiguous, revealing that something happened but without enough detail to determine if there was abuse or assault. Nonetheless, we used a consistent method based on our reading of thousands of tweets. The NPV and PPV of the trained machine learning estimates were good, based on our annotation. However, a different pair of annotators may have had slightly different estimates.

### Conclusions

Despite the noted limitations, our results highlight the magnitude of the mass sharing of personal experiences of sexual assault/abuse, filled with narratives of early life experience, which enabled the spread of the #MeToo movement and had a broad reach across Twitter. Further, these tweets and the archetypes presented here provide rich details to augment existing statistics captured traditionally from survey data and small in-depth studies of sexual assault and abuse survivors. The archetype provides a picture of what the public saw. The candid and revealing statements reminded followers and the public alike of the magnitude sexual violence and how it is often first experienced in early life and hidden. Therefore, the descriptive narratives could be used in public health survey development to assess whether there has been a change or a deeper public understanding of the prevalence, early life experience, and enduring trauma of sexual assault and abuse.

The summary of the content of tweets presented in this study highlights the initial conversation and demographics of participants in the conversation in the early stages of the #MeToo movement. Although our results present a snapshot of the public discourse on sexual violence and the initial participants, future work could examine the content and directions of the national conversation, which has since taken many directions and varies by populations.

## Appendix

Multimedia Appendix 1 Details of the selection process.

Multimedia Appendix 2 All versus novel tweets: example table.

Multimedia Appendix 3 Estimates of number, proportion, and reach of MeToo tweets from Oct 14 to 21, 2017.

Multimedia Appendix 4 Comparison of Twitter followers for abuse/assault and early experience sample against all twitter users.

## Abbreviations

API: application programming interface
LASSO: least absolute shrinkage and selection operator
NPV: negative predictive value
OLS: ordinary least squares model
PPV: positive predictive value
SVM: support vector machine
