# Supplementary material for: The #MeToo Movement in the United States: Text Analysis of Early Twitter Conversations
Source: J Med Internet Res. 2019 Sep 3;21(9):e13837. doi: 10.2196/13837 (PMC6751092; doi:10.2196/13837)
Supplement: Multimedia Appendix 1 [file jmir_v21i9e13837_app1.pdf]

**Multimedia Appendix 1: Supplemental Methods**

**Supplemental Methods 1: Detailed Data Extraction Description**

Several factors pertaining to obtaining Twitter Premium API data contribute to the discrepancy between the 12,337 novel English geotagged tweets and the subsample sample of 11,935 novel English geotagged tweets that we use in this study.

The Twitter Premium API has a maximum retrieval limit of 500 tweets per data request and each request costs money. Therefore, we had to optimize our requests. We decide to download tweets based on non-overlapping times in which they originated. This process led to the loss of some tweets during periods with more than 500 Tweets. For example, if we requested Tweet between 8pm-9pm on Oct. 15 and there were over 500 Tweets, say 637, we recovered only the first 500. The remaining 137 Tweets would have to be recovered in a separate request. Therefore, we examined the data to try to ascertain the time of last tweet downloaded to then send a new request with non-overlap times to fill in missing tweets. However, this process was inherently error prone. In some cases, we lost a few minutes and in others we overlapped in time. This led to both some duplicate Tweets and brief periods with missing data.

After data cleaning we had 11,935 non-duplicates tweets but based on the Twitter API counts there were 12,337 that should have met our search criteria. However, the counts data depend on the number of non-deleted tweets at the time of query. Twitter support documentation states, "The counts delivered through this endpoint reflect the number of Tweets that occurred and do not reflect any later compliance events (deletions, scrub geos). Some Tweets counted may not be available via data endpoint due to user compliance actions." We queried the counts before retrieving the data, so in addition to having brief periods with missing data, some small number of tweets may have been deleted by the users.

## **Supplemental Methods 2: Description of the Demographics Pro Algorithm**

Demographics Pro is a commercial service used to assess demographics and other characteristics of social media populations. We used their services to infer gender, age and race/ethnicity of the posters of revealed experiences. We provided Twitter handles, or screen names, of posters categorized as having revealed an experience of sexual assault/abuse and early life experience of sexual assault/abuse. Demographics Pro then provided us with a distribution of gender, age and race/ethnicity for the handles we shared and similar data for a random sample of 20,000 Twitter users in the US as a comparison.

Demographics Pro uses a series of proprietary machine learning algorithms to estimate or infer likely demographic characteristics of Twitter handles based on Twitter behavior/usage. Their predictions rely on signals from networks, consumption, and language use. They have created and validated their methodology against 300 million Twitter users. They require 95% confidence to make an estimate on a single demographic characteristic of a handle. Importantly, they do not return individual level data to researchers for us to make independent assessments of their algorithms, rather they return aggregates on the sample. This is to ensure privacy for Twitter users.

See http://www.demographicspro.com/views/demographics_aboutmeth.shtml for more details.
