# Supplementary material for: The #MeToo Movement in the United States: Text Analysis of Early Twitter Conversations
Source: J Med Internet Res. 2019 Sep 3;21(9):e13837. doi: 10.2196/13837 (PMC6751092; doi:10.2196/13837)
Supplement: Multimedia Appendix 2 [file jmir_v21i9e13837_app2.pdf]

Multimedia Appendix 2: All vs. Novel Tweets—example table

MeToo Tweets Examples	Novel MeToo Tweets Examples
“RT @500daysofMary: I was 13, he was 19. He lied to me and said he was 17. I was a child, but I was told it was my fault. #MeToo https://t.c… “	"#MeToo A ‘friend’ took ‘care’ of me after a party in college because I drank too much. Care = Rape I was 18. I trusted him.”[a, b]
“RT @womensmarch: To all the women sharing stories of sexual assault and sexual harassment, thank you for your bravery to speak up. You are not alone.”	“First time, 1995 when I was dead asleep and woke up to a man forcing himself on me. Too scared to fight, apparently NO wasn't enough. #MeToo" [a, c]
"RT @MarisaKabas: my entire twitter & facebook feeds are full of women i know saying #MeToo. Men, no matter what your history---just let this sink in."	“Every women in my family #MeToo has been raped. What are our national stats and how do we compare? #IHearYou" [d]
“RT@ @ColeLedford11:Every 98 seconds, someone is sexually assaulted. It’s time to let victims know they are not alone. #MeToo”	“The estimate of 30% of all girls/women sexually assaulted is probably too low.” [e]

Note: Tweets are presented in their original form as typed by the original poster.

[a] Clear example of first-person revelation of sexual assault or abuse

[b] Clear example of first-person revelation of early life experience of sexual assault or abuse

[c] Ambiguous example of early life experience because we do not know the age of the poster. Raters agreed that this was a case with an experience of sexual assault but disagreed on whether this was an early life experience.

[d] Ambiguous example of sexual assault tweet because if the poster is male and posting, this is a support tweet. If the poster is female, then it’s a revelation of rape. Raters disagreed on this case and searched Twitter. The poster was indeed male which is why we focus on first person revelations.

[e] Clear example of novel support tweet
