# Supplementary material for: The #MeToo Movement in the United States: Text Analysis of Early Twitter Conversations
Source: J Med Internet Res. 2019 Sep 3;21(9):e13837. doi: 10.2196/13837 (PMC6751092; doi:10.2196/13837)
Supplement: Multimedia Appendix 3 [file jmir_v21i9e13837_app3.pdf]

Multimedia Appendix 3: Estimates of Number, Proportion and Reach of MeToo Tweets from Oct. 14-Oct. 21, 2017

Tweet Type	Tweets Categorized by SVM	Percent of Novel English Language Geotagged Tweets	Est. of Novel English Language MeToo Tweets with Assault/Abuse	Lower Bound Estimates of Potential Reach [a]
Assault/Abuse	1287	11.4%	33,646	[5,955,342 to 34,251,628]
Early Experience	657	5.82%	17,176	[2,919,920 to 17,296,232]

[a] Reach is calculated by multiplying the estimated number of novel English language MeToo twees with revelation of sexual assault/abuse by the 25%-75% of range of the number of followers that poster of these events had.
