# Supplementary material for: The #MeToo Movement in the United States: Text Analysis of Early Twitter Conversations
Source: J Med Internet Res. 2019 Sep 3;21(9):e13837. doi: 10.2196/13837 (PMC6751092; doi:10.2196/13837)
Supplement: Multimedia Appendix 4 [file jmir_v21i9e13837_app4.pdf]

Multimedia Appendix 4: Comparison of Twitter Followers for Abuse/Assault and Early Experience Sample against all Twitter Users.

	Twitter Overall (%)	Abuse/Assault Sample (%)	Early Experience Sample (%)
**Followers**			
> 1,000 followers	17.6	15	15
101-1,000 followers	59.7	61.6	61.6
10-100 followers	21.9	22	21.9
< 10 followers	0.9	1.4	1.5

Note: Based on analysis of our analytical sample provided by Demographics Pro
